# Biophysical Characterization and Anticancer Activities of Photosensitive Phytoanthraquinones Represented by Hypericin and Its Model Compounds

**DOI:** 10.3390/molecules25235666

**Published:** 2020-12-01

**Authors:** Valéria Verebová, Jiří Beneš, Jana Staničová

**Affiliations:** 1 Department of Chemistry, Biochemistry and Biophysics, University of Veterinary Medicine & Pharmacy, Komenského 73, 041 81 Košice, Slovakia; valeria.verebova@uvlf.sk; 2 Institute of Biophysics and Informatics, First Faculty of Medicine, Charles University, Kateřinská 1, 121 08 Prague, Czech Republic; jiri.benes@lf1.cuni.cz

**Keywords:** natural photosensitive compounds, anticancer activity, hypericin, emodin, quinizarin, danthron, interaction, DNA

## Abstract

Photosensitive compounds found in herbs have been reported in recent years as having a variety of interesting medicinal and biological activities. In this review, we focus on photosensitizers such as hypericin and its model compounds emodin, quinizarin, and danthron, which have antiviral, antifungal, antineoplastic, and antitumor effects. They can be utilized as potential agents in photodynamic therapy, especially in photodynamic therapy (PDT) for cancer. We aimed to give a comprehensive summary of the physical and chemical properties of these interesting molecules, emphasizing their mechanism of action in relation to their different interactions with biomacromolecules, specifically with DNA.

## 1. Photodynamic Therapy

Photodynamic therapy (PDT) is part of photochemotherapy and requires the presence of a photosensitive substance (drug, PS), oxygen, and a powerful light source in the area of absorption of the PS used. The main requirements for activating the properties of a PS are its selective accumulation in tumor tissue, high intensity of absorption in the visible and near-infrared region of the spectrum, low level of dark toxicity, and absence of side-effects [1,2]. Selective accumulation and retention of PS in tumor tissues rather than in the surrounding healthy tissue lead to selective destruction of the tumor in PDT, while the surrounding healthy tissue remains intact. Such selectivity is one of the biggest advantages of this method, which may be substituted in some cases for chemotherapy, radiotherapy, or surgery in the treatment of cancer. Due to drug excretion [3] and redistribution, the effective therapeutic dose entering tumor cells is only a fraction of the administered PS. Administration of increased amounts of therapeutics is not possible because they have cytotoxic effects, which could cause significant toxicity in healthy cells. It is very important therefore to find alternative approaches, which increase the efficacy of the drug dose in the tumor and decrease the dose in healthy tissue [4]. Higher selectivity of PSs for tumor cells can be achieved by combining them with transport agents, which preferentially interact with tumor cells, ensure the selective accumulation of the drug within the diseased tissue, and deliver the desired therapeutic drug concentration to a targeted site in the patient’s body. Transport systems commonly used for photosensitizers are polymers, liposomes, oil emulsions, certain metals, some proteins, and carbon-based nanoparticles [5,6,7,8,9,10]. Stable and biocompatible transport systems with a long half-life in the blood are ideal. Selective drug delivery to tumor tissue, transport of nanoparticles containing a PS, and a tumor cell with a receptor is the objectives for achieving high selectivity and low drug concentration [11]. Several research groups have confirmed the hypothesis that one possible approach to achieving these goals is to prepare low-density lipoprotein (LDL)-based particles [12,13,14,15,16,17].

### 1.1. PDT in Cancer Therapy

PDT has been a promising, non-invasive method for the treatment of certain types of cancer for more than 25 years [18,19,20,21,22,23]; in addition, PDT has also been studied in various non-oncological applications; leishmaniasis [24], psoriasis [25,26], age-related macular degeneration [27,28], hyperplasia [27], restenosis [25], and in cardiology [29], urology [30], immunology [31], ophthalmology [28,32], dental medicine [33,34], and dermatology [35,36]. The combination of light and PS also shows promising results in the treatment of bacterial, fungal, parasitic, and viral infections [37,38,39,40].

**Figure 1:**
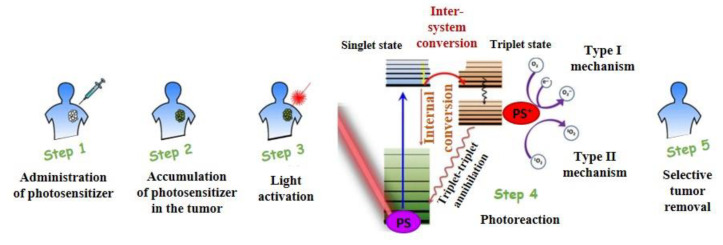
Scheme of photodynamic therapy (PDT; edited by [42,43]).

The basic process in tumor PDT is shown in the diagram in Figure 1. The photosensitizer is usually administered intravenously (systematically) or topically, and the administration of PS has also been studied orally, mostly in patients with extensive damage (not always caused by cancer) [1,41].

PS accumulates in the tumor tissue, which is subsequently irradiated with a light source of suitable wavelength, leading, in the presence of oxygen, to the formation of reactive oxygen species (ROS) and destruction of tissue and whole cells [1,41], while deactivation of important enzymes occurs and changes in the properties of biomacromolecules [44]. After activation of the photoactive substance by light and its interaction with molecular oxygen, singlet oxygen is formed. The singlet oxygen released in this photochemical reaction is highly toxic and can directly cause the death of tumor cells, through apoptosis, necrosis, or autophagy mechanism [45]. The release of singlet oxygen evokes oxidative stress, increases cytotoxicity, and DNA damage in cancer cells, modifies cellular metabolism, alters cancer cell death signaling pathways [46]. It also damages the blood vessels in tumor cells, resulting in indirect cell death through hypoxia (deficiency of oxygen) or aging (cell starvation). Diffuse distance of singlet oxygen obtained from photobleaching experiments has been appointed as 10–20 nm [47]. More recent time-resolved phosphorescence measurements show higher diffuse distance corresponding to 100 nm [48]. An explanation of this discrepancy was given by Hatz et al. [48] considering a fact that singlet oxygen behaves as a selective rather than reactive intermediate upon encountering other molecules in the cell.

Tissue damage depends on the depth of light penetration used to activate the PS. Membranes are damaged by cell death, triggering a number of inflammatory and immune processes, which cascade and cause the death of other tumor cells [49]. However, it was found out that singlet oxygen might cause membrane destruction directly. The cell membrane can be the main target of the singlet oxygen reaction for the bacterial membrane [50] and eukaryotic plasma membrane [51].

After surface irradiation PDT, the deeper connective tissues are only slightly damaged, so the patient’s body can begin to restore the structure and flexibility of the damaged cells, and subsequently the tissues [52,53].

### 1.2. Physicochemical Mechanism of PDT

The photodynamic effect can be induced by two mechanisms called Type I and Type II (Figure 1—Step 4). After photon absorption, the PS molecule goes from the ground state (S_0_) to the singlet excited state (S_1_). From this excited state, the PS can be returned to the ground state by energy emission through non-radiative and/or radiant processes (fluorescence). In its excited state, the PS can also spontaneously move from the singlet state S_1_ to the excited triplet state (T_1_) by means of the intersystem conversion process. In this state, the transition to the ground state through a phosphorescence process can occur [54].

The type I mechanism involves electron transfer reactions between the PS molecule in the excited states of S_1_ and T_1_ and the substrate. This process results in the formation of ionic radicals, which tend to react immediately with oxygen to form a mixture of highly reactive oxygen radicals, such as superoxide radical (**·**O_2_), hydrogen peroxide (H_2_O_2_), and hydroxyl radical (**·**OH), which oxidize a wide range of biomacromolecules [2,55].

The type II mechanism is characterized by energy transfer reactions between PS in the excited triplet state T_1_ and molecular oxygen, which is also in the triplet ground state (T_0_). These reactions cause the formation of singlet oxygen (^1^O_2_), which is able to rapidly oxidize cellular structures such as proteins, lipids, nucleic acids [56,57], and organelles leading to tumor cell death [58,59]. This also means that PDT may be a useful alternative treatment for cancer cells resistant to chemotherapy [60,61].

The reaction mechanism depends on the following conditions. First, the location of the PS is crucial because most of the ROS are highly reactive and cannot move far from the point of origin before disappearing. Second, the relative number of target biomolecules is important [43]. Davies (2003) calculated the percentage of ^1^O_2_ responses in leukocytes: protein 68.5%, ascorbate 16.5%, RNA 6.9%, DNA 5.5%, beta-carotene 0.9%, NADH/NADPH 0.69%, tocopherols 0.5%, reduced glutathione 0.4%, lipids 0.2%, and cholesterol 0.1% [62]. This means that the distribution of ^1^O_2_ may vary in different cell targets. 

Both mechanisms can occur simultaneously. Their proportional representation is significantly influenced by the PS, the substrate, the oxygen concentration, and the binding of PS to the substrate. In addition, the type II mechanism appears to be more efficient as it has a higher rate constant than electron transfer reactions (type I mechanism). As a result, energy transfer to other compounds that can compete with oxygen is less important, so type II is more often dominant [58,63].

### 1.3. PDT Applications

PDT currently occupies an increasingly important place in clinical medicine. Its most promising application is the treatment of small and superficial tumors, e.g., some types of skin, neck, stomach [64], lung [65,66,67], bronchial [68] or oral cancer [69], esophagus [70,71], bladder [71,72], brain [73], prostate and pleura [74] tumors, breast [75] but also skin disease [76], atherosclerosis, and viral diseases therapy [77], including AIDS [78]. 

The future of PDT lies in efforts to find or synthesize new photosensitizers with properties allowing their greater selectivity for tumor tissues, and to find new approaches for the specific localization of already-known PS in tumor cells. This should contribute to the wider and more effective application of PDT in clinical practice. Targeted PDT has several advantages they are important in participation in the treatment of oncological diseases. Some of them are listed below: high selectivity, negligible side effects, low level of complications during and after treatment, high quality of life for patients, diagnostics and therapy in one step, and lower financial costs [79]. It can be a convenient complementary therapeutic method to classical surgery, chemotherapy, radiotherapy, and independent limited diagnosis.

## 2. Plant-Derived Photosensitive Substances

Photoactive compounds occurring in medicinal plants with potential utilization in PDT have been found to be less toxic than synthetic agents. The reduction of side effects using natural PSs in cancer treatment is another advantage of this therapeutic approach. However, their clinical applications have been limited by several imperfections such as accumulation in tissues, a lack of chemical purity, or low penetration [80]. Muniyandi et al. [81] have published a comprehensive review article about the role of photoactive phytocompounds in PDT. Phototoxic effects, potential applications in the PDT of cancer of the main natural PSs groups (furanocoumarins, thiophenes, alkaloids, curcumins, polyacetylenes, and anthraquinones) have been described [81]. Our paper focuses on the four anthraquinones, of which hypericin is the most promising PS in the PDT of cancer. With regard to the hydrophobicity of studied anthraquinones, which is important for their penetration through membranes, the following types of PSs have been distinguished.

### Types of Photosensitive Substances by Hydrophobicity

PDT either uses chemotherapeutics commonly applied in chemotherapy, which must also be photosensitive, or new PSs are proposed. All photosensitizers (except uroporphyrin and photophrin), which have been proposed as drugs for use in PDT, interact more or less with serum proteins after intravenous administration. From the point of view of PDT, however, the interaction of the drugs with DNA is also quite important, as it is necessary to disrupt and stop the division of tumor cells, and this can be achieved only through their interaction with DNA [62,82]. In some cases, this interaction is not very significant (as these drugs have a greater affinity for proteins), so it is necessary to find a transporter that will help deliver the drug to the cell nucleus, thereby mediating the drug–DNA interaction [83,84]. Success in the treatment of cancer requires sufficiently hydrophobic drugs to cross the lipid membrane. For this reason, the hydrophobicity of drugs plays an important role in their distribution, metabolism, and excretion from the patient´s body. Due to these facts, we distinguish four groups of drugs with the ability to localize and accumulate in the tumor. Moreover, there are no rigid boundaries between these groups of drugs, and there is some overlapping between them, in some cases a continuous transition.

1. Hydrophobic PSs—compounds requiring the presence of transporters, such as liposomes or Cremophor EL, or Tween 80. They have the ability to localize in the inner lipid part of lipoproteins, mainly in LDL and high-density lipoproteins (HDLs), but also in very-low-density lipoproteins (VLDLs). This group includes phthalocyanines (ZnPC, C1A1PC), naphthalocyanines (isoBOSINC), tin-etiopurpurine (SnET2) [84], and hypericin [85].
2. Amphiphilic PSs—asymmetric compounds, which can be incorporated into the outer phospholipid and apoprotein layer of lipoprotein particles, e.g., disulfonates (TPPS_2a_, C1A1PCS_2a_), lutetium teraphyrin (LuTex), and monoaspartyl chlorine (MACE), which forms a barrier between albumin and HDL [84]. Emodin can be included in this group [86].
3. Hydrophilic PSs—drugs that predominantly bind to albumins and globulins, e.g., tetra-sulfone derivates of tetraphenylporfin (TPPS_3_ and TPPS_4_) and chloroaluminum phthalocyanine (C1A1PCS_3_ and C1A1PCS_4_) [84].
4. Intercalators—drugs that are used mainly in chemotherapy, which intercalate into DNA and are also photoactive, e.g., doxorubicin [87], daunorubicin [88], adriamycin [89], quinizarin [90], and danthron [91].

In this work, we focused in more detail on a very prospective PS in PDT of cancer hypericin. During a study of hypericin molecule incorporation into biomacromolecules (we focused on DNA) model compounds are using for simplification of the problem. Anthraquinones emodin, quinizarin, and danthron represent a significantly smaller part of the larger hypericin molecule with the same chemical groups. This fact facilitates the creation of a proper model for interaction between hypericin and biomacromolecules. Moreover, chosen hypericin derivatives themselves originate from medicinal plants, as the PS can be utilized in PDT and their anticancer effects are known. With respect to the above-mentioned sorting of PSs into groups, they can be representatives of highly hydrophobic (hypericin), mildly hydrophobic (emodin), and intercalating molecules (quinizarin and danthron). 

Scientists and physicians are currently working on how to increase the effectiveness of cancer treatment. One option that has been shown to be very effective is a combination of therapies (PDT and chemotherapy), which involve the direct or mediated interaction of anticancer drugs with DNA and other bioactive macromolecules (serum albumins, lipoproteins) [92,93,94]. Hypericin, emodin, quinizarin, and danthron are examples of anticancer drugs which are chemotherapeutics synthesized by medicinal plants and PSs, and which can be used, in PDT. The discovery of new natural drugs is very important because they have many benefits for the patients. Drugs derived from medicinal plants are less toxic to the body, their use poses less risk of adverse side effects, does not depends on them, they are suitable for all age groups of patients, and can be easily combined with conventional drugs, i.e., do not show contraindications.

## 3. Hypericin

**Figure 2:**
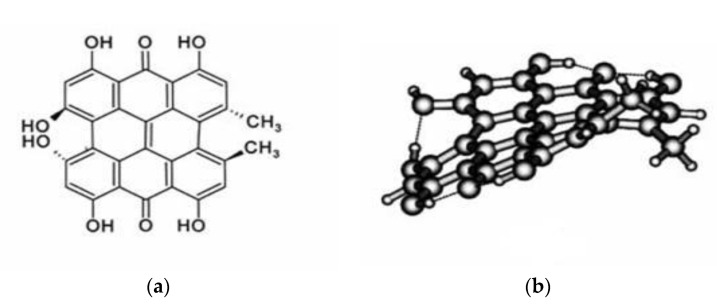
Two-dimensional (**a**) and three-dimensional (**b**) structure of hypericin [97].

Hypericin, a naturally occurring pigment, is found in certain plant species of the genus *Hypericum*. The most important representative is Saint John´s Wort (*Hypericum perforatum*) from the family *Hypericaceae*, a plant with golden-yellow flowers that grows to a height of 30–90 cm [95]. Hypericin has also been reported as occurring in some species of Australian insects [96]. Hypericin, 7,14 dione-1,3,4,6,8,12-hexahydroxy 10,11 dimethyl-phenanthrol [1,10,9,8-opgra] perylene (Figure 2) is an aromatic polycyclic dione, which exhibits a wide range of biological activities.

It has long been used to treat depression caused by a disorder of the brain neurotransmitters responsible for human moods [98,99]. Hypericin has antiviral [100,101] and antitumor activity [102,103,104,105] and is used to heal wounds, neuralgic sites, and hypertrophic scar [106,107,108]. Hypericin has been demonstrated to be very effective on bacteria [109,110,111,112,113,114]. Studies of hypericin´s antiviral activity confirm that it is known to effectively deactivate enveloped viruses, but it is ineffective against non-enveloped viruses. The antiviral activity of hypericin has been demonstrated against several types of viruses: herpes simplex virus, murine cytomegalovirus, sindbis virus [115,116], hepatitis B [117], anemia, some types of leukemic viruses [118], and HIV [119,120,121], so it has also been used in the clinical treatment of AIDS patients [122]. Hypericin activity depends to a large extent on the presence of light and oxygen. These two factors determine the extent of its antiviral activity and both antiviral and antitumor activities increase significantly after visible light irradiation [123].

Hypericin, together with two other hydroxyquinones, hypocrellin A and calphostin, is characterized by negligible dark toxicity, significant photocytotoxicity to tumor cells, intense absorption at higher wavelengths (>550 nm), higher selectivity for tumor tissues, and faster leaching than hematoporphyrins [124]. Hypericin is soluble in most solvents. In organic solvents (DMSO, ethanol) its solution is red; in basic media, it is green; and in aqueous solutions, it forms purple dispersed particles [125]. The absorption spectrum of hypericin in neutral organic solvents has two main transitions in the visible region, *S*_0_→*S*_1_ (500–600 nm) and *S*_0_→*S*_2_ (425–485 nm), and two intense absorption bands. Hypericin forms non-fluorescent aggregates in aqueous solutions. The absorption and fluorescence spectra of hypericin in hexane, chloroform, and toluene are similar to those in water, so it can be assumed that hypericin molecules also form aggregates in these solvents. An indicator of the formation of aggregates in solution is the absence of fluorescence [126]. Several physical and chemical properties of hypericin are shown in Table 1.

The photophysics of hypericin is very complicated, and some effort to interpret its photophysical and photochemical properties is necessary to elucidate its pharmacological and biological activities. Phenols are known to be good electron donors, while quinones act as electron acceptors. Hypericin contains both phenolic and quinone moieties. This structural characteristic places it in the group of amphi-electron compounds. The results of experiments suggest that hypericin has the potential to be a good oxidizing and reducing agent [128]. At present, the mechanisms leading to the selective accumulation of hypericin in tumor tissue compared to normal tissue are not known, nor the mechanism of its antiviral and antitumor action has been clearly determined. Thomas et al. proposed a mechanism of hypericin activity that depends on the presence of molecular oxygen, consisting of transferring energy from the excited triplet state of hypericin to the ground state of molecular oxygen, thereby generating oxygen in the singlet state, which is a highly-reactive molecule [129]. Singlet oxygen production has been theoretically confirmed [118] and experimentally demonstrated in organic solvents and lipid media [130,131,132]. Other studies suggest that although oxygen may play an important role in some cases, it is not always necessary for the antiviral activity of hypericin [133]. According to the work of the J.W. Petrich group, the origin of the photoinduced antiviral activity of hypericin may lie in its ability to produce a local pH drop in cells after irradiation [134]. The close relationship between the mechanism of hypericin antitumor activity and photoactivated local acidification in cells has been confirmed in experiments on tumor cell membrane experiments [102].

Hypericin is currently considered as a potential antitumor drug in PDT. It belongs in the group of highly hydrophobic PSs (AlogP = 5.04) [135]. Its cytotoxic and antitumor activity has been demonstrated, and its inhibitory effects on epidermal growth factor-R and a wide range of protein kinases (protein tyrosine kinase, MAP-kinase, and protein kinase C) [136]. The significant photosensitivity properties of hypericin together with its selective uptake in tumor tissues, especially in bladder cancer cells and its minimal cytotoxicity in the dark, are major positive indications for the clinical use of hypericin in the photodynamic cancer treatment [137]. Hypericin is a photosensitizer found in the endoplasmic reticulum, which, when activated by light, mediates the rapid emptying of calcium Ca^2+^ from the endoplasmic reticulum, which is linked to programmed cell death [138]. Although hypericin has been shown to be a potent inducer of cell death in PDT, some in vivo studies suggest that cells treated with PDT may activate rescue-signaling pathways, which may ultimately lead to tumor survival and recurrence [18,139].

Hypericin is a very good fluorescent probe for super-resolution microscopy [140]. Knowledge of the distribution of this molecule in the individual components of a living system is essential in detecting the modes of action of hypericin in biological organisms. The accumulation of hypericin in cells is determined by the diffuse and soluble properties of the molecule. It is known that after the initial accumulation in the cell membranes (20 min), an increased concentration of hypericin in the cell nucleus is observed with increasing time [141]. This indicates the possible interaction of hypericin with nucleic acids, the most essential component of the cell nucleus. Ultraviolet resonance Raman spectroscopy with 257 nm excitation was used to study nucleic acid-hypericin complexes. The excitation used amplifies the vibrational modes of nucleic acid bases. Resonance Raman spectra for complexes of polynucleotides with hypericin showed that hypericin preferentially interacts with purines at the N7 position, and this interaction is stronger in guanine than in adenine [142]. In addition, the spectra of the complex of hypericin with polyrG and calf thymus DNA, which were studied by means of surface-enhanced Raman spectroscopy, are dominated by bands corresponding to hypericin vibrations. Based on the spectra obtained, the importance of the guanine base in the interaction of hypericin with nucleic acid was confirmed. The spectrum of the polyrG complex contains changes similar to those in the spectrum of the DNA complex. Based on these results, it can be assumed that DNA induces changes in the stretching vibrations of the hypericin skeleton associated with the vibrations of the hydroxyl groups, suggesting that the hydroxyl groups of hypericin participate in the interaction with nucleic acid [143].

The mechanism of hypericin distribution in tissues, cells, and cellular organelles is also thought to be a relatively complex process, which is affected by hypericin concentration, incubation time, and properties of biological units. The bio distribution of hypericin in human organisms also depends on the mode of transport in the bloodstream. Hypericin binds to serum proteins, where lipoprotein particles appear to be a more efficient transporter than albumin. The interaction of hypericin with albumin is specific [144], while its interaction with lipoproteins is non-specific [145]. After binding with macromolecules (lipid structures, serum proteins, and sugars), hypericin forms biologically active monomers with an emission band around 600 nm, thus overcoming problems with its solubility in physiological solutions, which is very important in terms of its activity [97,137].

## 4. Emodin

**Figure 3:**
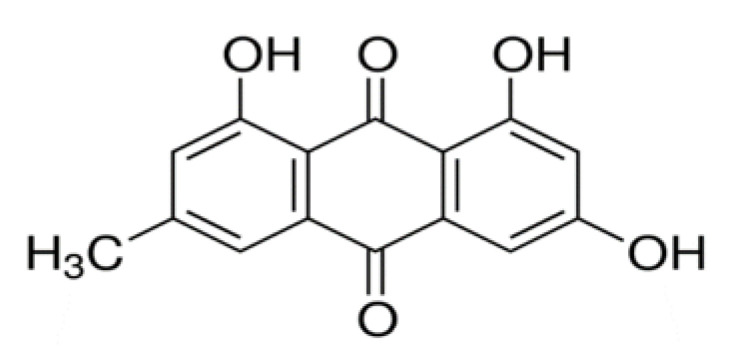
Structural formula of emodin.

Emodin, (1,3,8-trihydroxy-6-methylanthraquinone, Figure 3) is an anthraquinone derivate, a naturally-occurring pigment isolated from the underground part of the traditional Chinese medicinal plant *Rheum palmatum*, which is used mainly for its antitumor, immunosuppressive, antiinflammatory [146], antibacterial, diuretic, laxative [147], antiulcerogenic (antiulcer) [148], and vasorelaxant effects [149]. It is also found in other plant species, such as *Cassia*, *Aloe*, and *Rhamnus* [150,151,152,153,154]. In practice, it is available in the form of an orange crystalline substance. It is insoluble in aqueous media, and it forms aggregates (mostly in the acidic pH range). Emodin shows poor solubility in chloroform, ether, and benzene, but is highly soluble in DMSO and ethanol.

The antiviral properties of emodin have also been confirmed, mainly on enveloped viruses. This property of emodin is related to its high affinity for the phospholipid membrane, in which it inhibits hydrophobic interactions between individual hydrocarbon chains [155]. Emodin has been proposed as a major precursor of the microbial metabolic pathway in the isolation of hypericin [156,157]. It is a redox catalyst and plays an important role in many processes, such as electron transport, photosynthesis, or cellular respiration [158]. Its effect on cell death has been widely studied both in the dark and after light activation [155,159]. The stimulatory effects and the prokinetic effect of emodin on gastrointestinal smooth muscle have been described in several studies [147,160]. In recent years, its effect on the contractility of smooth muscle cells has been studied [161,162].

In addition to the pharmacological properties mentioned above, emodin also has toxicological effects. Under aerobic conditions, it is photolabile in visible light and phototoxic in vitro and it can cause testicular toxicity in mice leading to hypospermatogenesis [163,164]. It inhibits protein kinase casein kinase II (CK2). Genetic disorder of the catalytic subunits of CK2 protein kinase leads to changes in sperm shape in mice during spermatogenesis and is also responsible for male infertility [165,166]. Several authors have studied the interaction of emodin with DNA [167,168,169]; for example, it causes the formation of DNA double-strand breaks by stabilizing topoisomerase II-DNA cleavage complexes and inhibiting ATP hydrolysis by topoisomerase II [170]. Emodin has a high binding affinity for serum proteins and is known to bind non-covalently to DNA in intact cells in the presence of serum proteins [159]. The interaction of emodin with proteins has been studied using several techniques surface-enhanced Raman spectroscopy, NMR, fluorescence spectroscopy, circular dichroism, and the stopped-flow method [171,172,173,174]. The formation of emodin–protein complexes is very important with regard to understanding the mode of drug delivery and transport in tumor cells [174]. Emodin belongs in the drug of amphiphilic PSs with mild hydrophobic properties (Alog P = 2.568) [135]. It has an anticancer effect on some types of human liver and lung tumors. However, the molecular mechanisms of emodin-mediated tumor regression have not been precisely defined [150]. Emodin has specific antineuroectodermal tumor activity in vitro and in vivo [175]. It suppresses tyrosine kinase activity on HER-2 breast cancer cells, inhibits the transformation of the phenotype of these cells, and can affect androgen receptors directly by inhibiting cell growth in prostate cancer cells [146]. The antitumor activity of emodin on the human chronic myeloid leukemia cell line K562 has also been demonstrated in vitro and in vivo [176]. Emodin is toxic for glioma cells by inhibiting their proliferation and inducing apoptosis of C6 cells [177]. It is known to increase the sensitivity of tumor cells to chemotherapeutics, but the mechanism of the emodin-mediated chemotherapeutic effect on cancer cells has been the subject of intense study for many years [150,178]. Important physical properties of emodin are listed in Table 1.

## 5. Quinizarin

**Figure 4:**
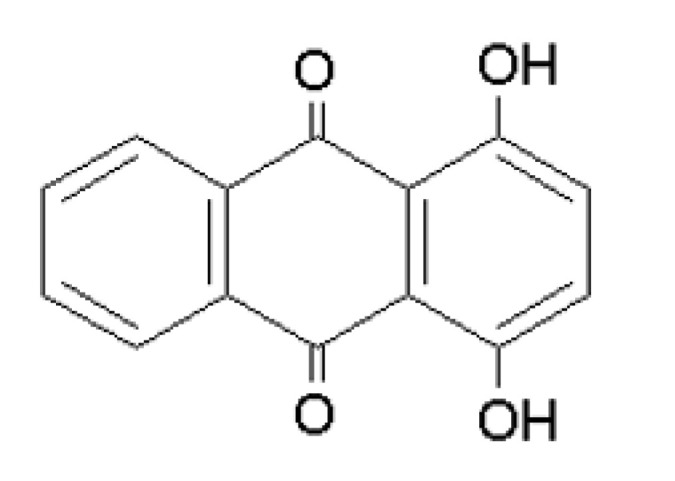
Structural formula of quinizarin.

Quinizarin (1,4-dihydroxyanthraquinone, Figure 4) is a polycyclic aromatic hydrocarbon containing two opposite carboxyl groups (C=O) at positions 9,10. It takes the form of a yellow crystalline substance and occurs in nature in plants (*Aloe*, Cascara Sagrada, Senna, and rhubarb), fungi, some lichens, and insects [179]. Quinizarin is soluble in basic solutions, acetone, chloroform, and DMSO. It is almost insoluble in aqueous solutions, but its solubility increases with increasing temperature. It is used as a pesticide as a fungicide and as an additive in lubricants [180]. In addition, it is used as a colorant in the food, textile, dyeing, and photographic industries [181]. Quinizarin inhibits HIV proteinase [182] and acts as a mutagenic agent on some mammalian bacteria [183]. Immunological analysis studies have shown that it stimulates the P450 enzyme in rat epithelial and liver cells [184].

The structure of quinizarin has been studied using several spectroscopic methods, such as fluorescence measurements [185], resonance Raman and infrared spectroscopy [186], and X-ray structural analysis [187]. It has planar C_2v_ molecular symmetry and crystallizes in a monoclonal system. Quinizarin is a highly fluorescent substance. The fact that it is almost insoluble in water significantly complicates the application of Raman spectroscopy in its study. However, infrared FT Raman and surface-enhanced Raman spectroscopy can also be used to characterize it in aqueous media [188]. 

Quinizarin is the simplest model molecule of a chromophore typical of some biologically and pharmaceutically significant compounds, including the antitumor anthracycline antibiotics doxorubicin, daunorubicin, and adriamycin, which are used in antineoplastic therapy. The quinizarin-like quinoid moiety is probably responsible for the cytotoxicity and cardiotoxicity of anthracycline drugs [180]. It is thought to be able to intercalate into DNA [189,190]. We can classify it among the photosensitive intercalators with low hydrophobicity (AlogP = 2.324), which could be applied in PDT [135]. The physical properties of quinizarin are shown in Table 1.

## 6. Danthron

**Figure 5:**
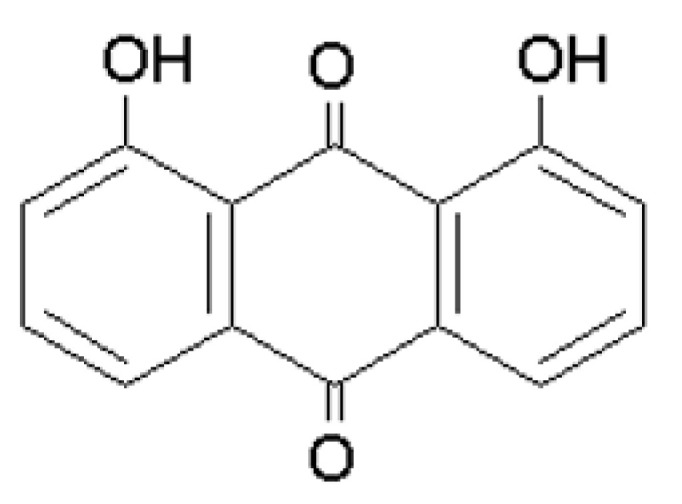
Structural formula of danthron.

Danthron (1,8-dihydroxyanthraquinone or chrysazine, Figure 5) is a hydroxyanthraquinone that occurs wild in nature found in many plants but also in some insect species (e.g., *Pyrrhalta luteola* larvae). It is very often isolated from dry leaves and stems of the plant *Xyris semifuscata* growing in Madagascar [191]. It is commercially available in the form of an orange, red, or red-yellow crystalline powder. Like quinizarin, it is soluble in basic solutions, chloroform, and DMSO. It is practically insoluble in water. Danthron and quinizarin are derivatives of emodin and hypericin. Danthron is part of the anticancer drug aclacinomycin. It is thought to intercalate into DNA and therefore can be used as an example of a low hydrophobicity intercalator (AlogP = 2.324) in PDT. However, it can also be a carcinogen, causing the development of adenoma or adenocarcinoma of the colon and increased incidence of liver cancer cells [191,192]. Danthron is one of the hydroxyanthraquinone derivatives causing topoisomerase II inhibition [193], which is involved in various cellular processes, including chromosome segregation [194], and it is essential for maintaining genome stability [195].

Danthron is used as a laxative [159,196]. It is currently applied as an antioxidant, a fungicide to control powdery mildew, and has an irreplaceable role in the research into anticancer agents. Together with emodin, it is the basic structure of aglycones, which are naturally occurring laxative glycosides [191]. Danthron shows mutagenic activity [197] and indicates mutations in cells of the lymphatic system [192].

Its structure has been studied using fluorescence, resonance Raman and infrared spectroscopy. The danthron crystal is tetragonal and its planar molecule has an asymmetric structure with two different intramolecular O…..O distances. The optical properties of danthron can be discussed in terms of C_2v_ pseudosymmetry [188]. Danthron and quinizarin form two intramolecular hydrogen bonds, which cause small changes in the structure of anthraquinone molecules. The presence of two hydrogen bonds in quinizarin allows for double proton transfer, which can take place in two steps or a one-step process. Conversely, the geometry of the danthron molecule allows only single proton transmission. The structure of danthron and quinizarin has been intensively studied and experimentally determined. For quinizarin, X-ray structural analysis identified the values of the distance O…..O and O…..H bounds, which are 2.57 and 1.77–1.79 Å respectively. This technique helped to define the O…..O distance for danthron and setting its value at 2.49 Å, but it did not provide any information about proton binding [198]. Table 1 shows characteristic physical properties.

## 7. Hypericin and Its Derivatives Interaction with DNA

In recent years the interaction of hypericin, emodin, quinizarin, and danthron with biomacromolecules has been studied, especially with DNA, LDL, and human serum albumin (HSA) has been investigated [199,200,201,202]. The results of our studies in this area supplement the scientific knowledge on this subject. The measurements indicate that PSs incorporate into DNA, LDL, and HSA, but interact most easily with LDL particles. Distribution of molecules has been studied at two different conditions: (i) DNA–ligand complexes were exposed to the presence of free LDL particles or HSA molecules, respectively and (ii) LDL– or HSA–ligand, respectively, complexes were in the presence of free DNA molecules. From measurements at the first conditions, it is clear that while the fluorescence intensity for the DNA-hypericin complex increases with the addition of LDL to the solution, a less pronounced effect can be observed for the other PSs, DNA–emodin, DNA–quinizarin, and DNA–danthron. Similarly, HSA molecules added to the solution slightly increase the fluorescence intensity in the DNA–hypericin complex. In a competitive environment, they redistribute and rebind from DNA macromolecule to LDL (hypericin) or HSA (emodin, quinizarin, and danthron). In contrast, the fluorescence intensity did not change for the DNA–emodin complex, and a slight decrease in fluorescence has been observed in the DNA–quinizarin and DNA–dantron complexes. The presence of free DNA leads to a decrease in the fluorescence intensity of the LDL–emodin, LDL–quinizarin, and LDL–danthron/HSA–quinizarin and HSA–danthron complexes. For LDL–hypericin and HSA–hypericin complexes, an increase in fluorescence with the addition of DNA was recorded. The fluorescence intensity of the HSA–emodin complex remains unchanged [135]. It is noteworthy that the quenching of fluorescence in the presence of DNA has been observed for intercalating molecules [190]. These results allowed us to state that the molecules of the studied PSs can be extracted from DNA by HSA molecules rather than LDL particles, with the exception of hypericin, where there is a more significant binding from DNA in the presence of LDL particles. Experiments in fluorescence analysis of native and denatured DNA were designed to determine if and how the interaction of hypericin, emodin, quinizarin, and danthron with native and denatured DNA differed. Significant changes were detected for quinizarin and danthron, where fluorescence increased significantly in the presence of denatured DNA. It can be assumed that DNA denaturation increased the distances between the individual quinizarin and danthron molecules (there is a decrease in fluorescence quenching) and helped to better visualize the molecules inside the DNA. This applies in particular to molecules that intercalate into the DNA structure, i.e., quinizarin and danthron. For hypericin and emodin, it has been noted only a slight change in the intensity of fluorescence in the presence of denatured DNA, respectively from which it can again predict a different interaction mode of these PSs with DNA [135].

As a representative of highly hydrophobic PSs, hypericin binds to DNA by means of a large groove [203,204]. A binding constant, whose value was also determined, is 4.0 × 10^4^ L/mol [204]. Emodin, with mild hydrophobic properties, interacts with DNA by binding into a small groove. This interaction is characterized by binding constant 8.1 × 10^4^ L/mol. These ligands are incorporated into the DNA groove with hydrophobic or hydrogen bounds [204]. An intercalating mode of interaction with DNA has been confirmed for quinizarin and danthron, which is very clearly supported by atomic force microscopy measurements. Quinizarin and dathron cause DNA to unwind due to intercalation, increasing the contour length of linear DNA by 10%. By the action of natural photosensitizers studied by us, more rigid molecules of linear DNA have been detected. It is known that this is not only the type of reaction they are involved in. Quinizarin and danthron can also bind to the surface of DNA molecules by means of electrostatic forces. The size of the binding constant of this interaction type for both PSs is 1.1 × 10^4^ L/mol [190].

Cell experiments have been performed with the aim of better understanding the mechanisms of the interaction of hypericin, emodin, quinizarin, and danthron with DNA. Success in the treatment of cancer requires sufficient hydrophobic drugs to cross the lipid membrane. The hydrophobicity of drugs therefore plays an important role in their distribution, metabolism, and excretion from the patient´s body. The main barrier preventing the interaction of nuclear DNA with anticancer drugs is the nuclear envelope. On the other hand, the detoxification process is accompanied by a decrease in drug concentration during the active outflow of these drugs. P-glycoprotein, a member of the transport protein family, is involved in this process [205]. The function of these proteins is to remove toxins from cells [206], so it is possible to regulate drug resistance by manipulating P-glycoprotein activity [207,208]. Another approach to preventing cancer cell resistance to drugs could be based on photochemical activation at a well-defined site/organelle [209]. With this in mind, the intracellular interaction of the drugs discussed here with DNA directly in the nucleus was studied using selective photoactivation and regulation of P-glycoprotein function. Selective photoactivation facilitates entry of emodin, quinizarin, and danthron molecules into the nucleus of tumor cells by means of passive diffusion (this phenomenon has not been observed in the case of hypericin). Inhibition of P-glycoprotein may increase the intracellular concentration of the molecules studied and aid their interaction with DNA. Inhibition of P-glycoprotein stimulates the passive transport of these molecules to the nucleus of tumor cells. In addition, emodin is known to significantly promote the entry of other drugs into the nucleus of tumor cells, with the exception of strongly hydrophobic ones (hypericin-like), which are characterized by a high binding affinity for P-glycoprotein [135].

The treatment of tumor cells with natural photosensitive drugs significantly depends on the degree of hydrophobicity, lipophilicity, and DNA intercalation properties of the drugs used. 

## 8. Conclusions

Cancer is a group of diseases characterized by abnormal and uncontrolled cell growth, which can affect a particular tissue or organ in a very aggressive manner and are able to spread to other parts of the body, they can metastasize. Cancer can be treated by classical therapeutic procedures (surgery, chemotherapy, and radiotherapy) and modern, very promising therapeutic methods. These include PDT. These studies show that the most effective way to treat cancer is to combine PDT with traditional therapies, respectively in some cases, PDT can even replace conventional treatment. It is very likely that PDT will be used in the future as the therapeutic approach in the treatment of specific cancers and non-cancerous diseases. This method minimizes the side effects of traditional therapeutic methods, is less invasive, more effective, and affordable. The condition for the successful treatment of cancer with PDT is the disruption of DNA macromolecule, which stops the division of tumor cells. Therefore, it is necessary to find suitable drugs that interact with DNA directly, respectively mediated by transporters that transport them to the nucleus of the tumor cell. A great advantage of newly discovered drugs is that they are natural, easier to prepare, and do not burden the patient´s body as much as synthetically produced drugs. The success of cancer treatment is highly dependent on the application of hydrophobic drugs, which can more easily cross the lipid membrane of tumor cells. Therefore, the interaction of drugs with different hydrophobicity with DNA macromolecule is the subject of many studies. 

## Figures and Tables

**Table 1:** Physical and chemical properties of selected photosensitizers (hypericin, emodin, quinizarin, and danthron).

Photosensitizer		Hypericin	Emodin	Quinizarin	Danthron
Hydrophobicity		high	amphiphilic with mild hydrophobicity	low	Low
AlogP		5.040	2.568	2.324	2.324
Absorption maximum in DMSO λ_max_ (nm)		560 600	440	475	430
Fluorescence maximum λ_exc._ (nm)		603	520	540	575
		560	440	475	430
Dissociated form (nm)	PS^0^	560	440	475	430
	PS^1-^	600 [127]	480	560	475
	PS^2-^	650 [127]	525	595	500
	PS^4-^	630 [127]			
	PS^6-^	640 [127]			
Dissociated constant	pKa_1_	2 [127]	7.2	11.3	10.5
	pKa_2_	7.8 [127]	10.6	12.7	12.9
	pKa_3_	11.5 [127]			
	pKa_4_	13 [127]			
